# Patients and Health Professional's Perspective of Functional Mobility in Parkinson's Disease

**DOI:** 10.3389/fneur.2020.575811

**Published:** 2020-10-27

**Authors:** Raquel Bouça-Machado, Nilza Gonçalves, Inês Lousada, Maria A. Patriarca, Patrícia Costa, Raquel Nunes, Susana Dias, Ana Castro Caldas, Anabela Valadas, Patrícia Pita Lobo, Leonor Correia Guedes, Mário M. Rosa, Miguel Coelho, Joaquim J. Ferreira

**Affiliations:** ^1^Faculdade de Medicina, Instituto de Medicina Molecular, Universidade de Lisboa, Lisbon, Portugal; ^2^CNS-Campus Neurológico, Torres Vedras, Portugal; ^3^Department of Neuroscience and Mental Health, Neurology, Hospital de Santa Maria, Centro Hospitalar Universitário Lisboa Norte (CHULN), Lisbon, Portugal; ^4^Laboratory of Clinical Pharmacology and Therapeutics, Faculdade de Medicina, Universidade de Lisboa, Lisbon, Portugal

**Keywords:** functional mobility, Parkinson's disease, focus groups, concept, walking aids

## Abstract

**Background:** Functional mobility (FM) is the person's ability to move to accomplish daily living tasks and activities. FM limitations are common in Parkinson's disease, increase with disease progression, and can be highly disabling. Although several studies in Parkinson's disease (PD) field use this concept, only recently, a formal definition has been proposed.

**Objective:** We aimed to explore patient's and health professional's perspectives of FM in PD.

**Methods:** A focus group methodology has been used. Four focus groups, with a total of 10 patients and 10 health professionals, were performed. Six patients were early stage and four advanced stage. The health professional's group was composed of five neurologists and five physiotherapists. The suitability of the new concept, the impact of FM limitations in PD patient's daily routine, and the potential benefit of walking aids have been discussed.

**Results:** All participants were able to provide a spontaneous definition of FM, matching with the proposed concept. All agreed that PD affects patient's FM, increasing the limitations with disease progression, and with the existence of a serious prejudice with walking aids that hinders its use. Early-stage patient's perspective seems to be more in line with neurologist's perspective, while the views of advanced-stage patients were closer to physiotherapist's views.

**Conclusion:** FM concept was considered as intuitive and useful. FM limitations have an important physical and social impact in the advanced stage of the disease. Although patients and health professionals acknowledge walking aid's benefit improving patient's FM, the prejudice associated with this type of tools limits its recommendation and use.

## Introduction

Parkinson's disease (PD) is a complex and fluctuating neurodegenerative disorder associated with the presence of motor and non-motor symptoms, which can be very disabling and highly affect patients' quality of life (1). Despite an optimal disease management, many of these symptoms improve only partially and aggravate with disease progression, resulting in recurrent falls, reduced mobility, and loss of independence (1–3).

Functional mobility (FM) is the capacity of people to move from one place to another, in order to participate in the activities of daily living (ADL) at home, work, and in the community. This concept includes movements like standing, bending, walking, and climbing and contributes greatly to the subject's health-related quality of life (4).

In PD, both motor and non-motor symptoms contribute to the appearance of FM limitations. Although poorly defined, this concept has been frequently used in PD research. Recently, due to its frequent misuse, there is a need to clarify and to establish a formal concept of FM to be applied to PD (5).

The present study aims to explore, through a focus group methodology, PD patients and health professional's perspective on the proposed concept of FM, exploring also the impact of FM limitations in patient's daily life and the strategies to deal with it. We hope to clarify the suitability of the new concept of FM in PD and to promote a more holistic and functional approach to the patient's needs.

## Methods

### Study Design and Patient's Recruitment

A focus group methodology was used. Four focus groups were undertaken, two with patients (early and advanced disease stage) and two with health professionals (physiotherapist and neurologist—movement disorders specialists). Patients were included if they had the following: (1) PD diagnosis, according to the Movement Disorders Society clinical diagnostic criteria; (2) a Hoehn Yahr (HY) stage between I and IV under dopaminergic medication (MED ON); (3) the ability to communicate with the investigator and to understand and comply with the requirements of the study; and (4) the ability to provide written informed consent to participate in the study. Patients were excluded if they have been diagnosed with an atypical parkinsonism.

Health professionals were included if they work regularly with the PD population for at least 1 year. Participants were recruited from CNS—Campus Neurológico, a specialized movement disorders center (Torres Vedras, Portugal), and from the Deep Brain Stimulation surgery waiting list of the Movement Disorders outpatient clinic of a tertiary university hospital (Hospital Santa Maria, Lisbon, Portugal). The CNS Local Ethical Committee approved the study (Ref. 04-2018) and all participants provided written informed consent.

### Focus Groups

All participants that fulfilled the inclusion criteria were invited to participate. Information about the objectives, duration, procedures, and voluntariness was provided and the informed consent was obtained. Demographic and clinical data were collected for each PD patient. Patients were assessed in “ON” state medication. To define early and advanced PD, the presence of motor complications with impact in patient's daily life, assessed through MDS-UPDRS part IV, was used.

The focus groups followed a semi-structured script, including questions concerning patients and health professional's thoughts on the concept of FM, the impact and strategies to deal with FM limitations in daily life, and on the role of walking aids (Appendix 1).

Each focus group took up to 90 min (75 min to focus group questions and 15 min to close). At the beginning of each interview, participants were reminded of the purpose of the study and guaranteed confidentiality. Participants were encouraged to interact with each other, with the author intervening solely to keep the discussion on the topic and to encourage the more reserved members of the group to speak.

The focus group was recorded, with the agreement of all participants.

### Data Analysis

The audio recordings were transcribed and read until it reached an overall understanding.

Transcripts of the focus groups were divided into meaningful categories and themes. In a second step, a thorough read of the data was performed to ensure the identified themes were evident and a true reflection of the data was captured. Researchers moved back and forth in a reflexive process until consensus was reached.

Descriptive statistics were used for demographic, clinical, and therapeutic data.

## Results

Twenty participants were included in the study: six early-stage patients, four advanced-stage patients, five physiotherapists, and five neurologists. The mean age of patients was 68.0 ± 9.9 years (71.7 ± 9.0 in early stage and 60.7 ± 8.3 in advanced stage), with a mean disease duration of 8 ± 5.2 years (7.0 ± 6.1 in early stage and 10.0 ± 3.0 in advanced stage) and a mean Hoehn and Yahr score of 2.2 ± 0.4 (2.0 ± 0.4 in early stage and 2.5 ± 0.6 in advanced stage) (Table 1).

**Table 1:** Demographic and clinical data.

	**All patients (*n* = 10)**	**Early-stage group (*n* = 6)**	**Late-stage group (*n* = 4)**
Gender, M/F	7/3	3/1	4/2
Age at onset, mean years (SD)	68 ± 9.9	71.7 ± 9.0	60.7 ± 8.3
Disease duration, mean years (SD)	8 ± 5.2	7.0 ± 6.1	10.0 ± 3.0
% Tremor as first symptom	60%	50%	66.7%
MDS-UPDRS Part II, mean (SD)	12.4 ± 8.1	8.0 ± 2.3	21.43 ± 8.5
MDS-UPDRS Total score, mean (SD)	62.4 ± 23.6	61.0 ± 26.8	90.7 ± 18.3
HY, mean (SD)	2.2 ± 0.4	2.0 ± 0.0	2.5 ± 0.6

Patients in the early-stage group were autonomous, with an active lifestyle and/or exercise maintained through their professional job. Patients in the advanced-stage group were almost all retired, had less autonomy, and need more family support. For those who were employed, working conditions have been adapted to their specific needs.

Health professionals' experience with PD varied between 1 and 5 years in the physiotherapist group and between 5 and 20 years in the neurologist group. All neurologists were movement disorders specialists and all the physiotherapists worked in a specialized movement disorders center.

### The Concept of Functional Mobility

All groups were able to present a spontaneous definition of FM that matches with the one used by the authors. All agree that FM reflects the difficulties of PD patients in daily life (Table 2, Appendix 1).

**Table 2:** Key aspects mentioned by the four groups about the concept of FM.

**What does the concept of functional mobility suggest?**	
**Early-stage patients**	**Late-stage patients**
• Ability to move • What we do in daily life • Easy performing tasks • The functionality of my mobility is impaired • Something that never worried me	• Autonomy in daily life • Not needing others • It's getting out on the street without anyone noticing that I have Parkinson's • Wanting to do and look like you don't know how • Its dressing and move in bed
**Physiotherapist**	**Neurologists**
• Movement to perform a function • Daily life • Functionality • Functional movement • Different degrees of limitation	• Ease to displacement • Move to a goal • Movement to perform a task • Autonomy • Related with the WHO concept of Disability. The opposite of impairment.

#### Early-Stage Group

Early-stage PD patients associate the concept of FM with the ability to move and with easy performance of daily life tasks. For this group of patients, FM is something that will not worry them in their actual state.

#### Advanced-Stage Group

Advanced-stage PD patients associate FM with autonomy in daily life and with not being noticed by others in a public environment. Dressing and turning in bed were mentioned as activities related to FM.

#### Physiotherapist Group

Physiotherapists described FM as the movement for a function or the ability to accomplish the daily tasks important for the subject, even with limitations.

#### Neurologist Group

Neurologists described FM as the movement needed to perform a task regardless of how you do it and also as something that includes purposed displacements and transfers. For them, the concept of FM is close to the World Health Organization (WHO) concept of disability, as opposed to impairment, and should not be limited by the existence of displacement. In their opinion, the key aspect is the intention to accomplish a task or achieve a goal.

Neurologists highlighted the importance of having an operationalized concept of FM. In their opinion, this outcome may express better patient's perception of their overall health status and may help to adopt a more patient-centered approach. They also suggested FM as a potential useful outcome for the rehabilitation field.

### The Impact of FM Limitations in Patient's Lives

#### Early-Stage Group

Early-stage PD patients mentioned having more difficulty in some specific tasks (e.g., going down the stairs), mainly the need more for time to complete their usual tasks. In their opinion, except for direct family members and close friends, their FM limitations were not noticed by others. This group was not able to identify the best therapeutic strategy to deal with FM limitations. They hypothesize that exercise may be one of them, based on their experience of its benefits (Table 3, Appendix 1).

**Table 3:** Key aspects mentioned by the four groups about the impact of FM limitations on the patient's life.

**What is the impact of FM limitations on the patient's daily life?**	
**Early-stage patients**	**Late-stage patients**
• A higher difficulty to perform some tasks but mainly a slower rhythm • Friends and distant family are unaware • Close family refers a slowdown, difficulties in tasks like buttoning • Exercise, cognitive training are efficacious strategies to deal with FM limitations	• Clear perception of FM limitations associated with the disease • The most limiting factor of activities of daily living • The “OFF” periods are the worst moments of the day • Look for strategies to minimize the symptoms of the disease • Feel ashamed for drawing others' attention
**Physiotherapists**	**Neurologists**
• First limitations: stand up from a chair, get out of the bed or from the car • Associated with the stage of the disease • Initial devaluation, followed by sadness and frustration • In physiotherapy sessions patients learn how to deal with the limitations. Some patients find their own strategies.	• Vary from patient to patient, according lifestyle and tolerance with himself • Patients develop their strategies to overcome limitations until the moment they stop working • Sometimes the perspective of the impact of limitations and treatment goals between a patient and a neurologist does not coincide. The perspective between patient and caregiver is also different.

#### Advanced-Stage Group

Advanced-stage PD patients acknowledge to have limitations in FM and consider them the main limiting factor of daily activities, especially in “OFF” periods of medication. They refer that this type of limitation frequently draws other's attention to them, making them feel ashamed. Patients try to avoid these situations through social isolation or finding strategies to mask the signs of the disease. According to their perspective, family and closest friends are usually supportive, while friends and colleagues have more difficulties understanding the fluctuations of the disease. This usually contributes to social isolation and a higher burden to the family members. Medication adjustments, based on patient's priorities, and the use of walking aids were spontaneously referred as strategies to overcome daily life difficulties related to FM.

#### Physiotherapists

Physiotherapists associated the onset of FM limitations with disease progression. According to their experience, the first FM limitations, mentioned to or noticed by the physiotherapist, are getting up from a chair and getting out of bed or from the car. In physiotherapist's perception, patients start by devaluating these limitations, progressing to a feeling of sadness and frustration.

The importance of physiotherapy sessions to maintain PD patient's functionality in daily routine was highlighted, and the importance of patient's education and movement strategy training to overcome FM limitations was emphasized. It was referred that some patients have more difficulty learning due to the feeling of frustration or due to a higher negative emotional burden. In the physiotherapist's perspective, the collaboration of the psychology team is important in these cases. It was also referred that pharmacological interventions enhance the results of physiotherapy interventions, whereby this group supports that the management of PD FM limitations should be a joint work of the multidisciplinary team.

#### Neurologists

In neurologists' opinion, the interference of FM limitations depends on the patient's characteristics, such as affected side, expectations, and lifestyle (active, retired). Some patients, less demanding with themselves, seem to tolerate disability better.

According to neurologists, patients usually self-manage FM limitations until they can no longer do it. They develop their own strategies, such as wearing button-free clothes and shoes without laces or getting up early to be able to perform all the necessary tasks. It was referred that these limitations and strategies are not always noticed by the neurologist who follows them in the consultation. Neurologists also underline that patient's and caregiver's perspectives differ on this topic.

### The Use of Walking Aids

#### Early-Stage Group

For early-stage patients, the ability to complete a task and performing it successfully were the aspects they valued most in their daily lives, at the expense of the time needed.

The regular use of walking aids is not considered by this group of participants. They believe that good monitoring by specialized professionals and easy access to information about the disease are enough. Some mentioned to have used Nordic walk sticks to perform exercise and found it useful. All were open and suggested the development of technological devices that help them with disease-related problems, such as a device that reminds them to correct their posture. When asked about the key requirements of walking aids, it was mentioned the need for softeners to smooth the gait and the ability to adapt to different surfaces, to be light, and to have handles that allow the use of hands (Table 4, Appendix 1).

**Table 4:** Key aspects mentioned by the four groups about the use of walking aids.

**The use of walking aids**	
**Early-stage patients**	**Late-stage patients**
• The ability to complete a task successfully is the aspect more valuable. The time is no longer a priority when you know you have PD. • The use of walking aids depends on the needs of each patient. PD don't need this type of solutions. A good management of the disease, prevention and education by a specialist are more appropriated. • Patients were open to the use technological devices or Nordic sticks. • Due to the lack of experience, patients only mentioned suggestion for Nordic sticks. They mentioned the existence of shock absorbers to smooth the gait, tips adapted to different types of surfaces, light and with handles that allow to open the hands.	• Patients try to delay the use of walking aids through medication adjustments. • The patients used walking aids, by their own initiative, to get down, get up or when the gait was unstable. They did not have any period training. Falls occurred. • Due to the existence of “ON” periods in which they have acceptable functionality, they do not consider the use of permanent walking aids. • Patients express some reluctance to use walking aids due to the associated social stigma. • A bad experience with walking aids, without training or adaptation period, creates an insecurity that conditions future uses.
**Physiotherapists**	**Neurologists**
• The presence of imbalances and an increased risk of falling are the first warning signs for the need of walking aids. • They are usually faced in a negative way, as a sign of disease progression and a greater level dependence. • The fear of falling helps accepting the recommendation of a walking aid. • The choice of a walking aids should be personalized.	• According to the patient's clinical characteristics. • This recommendation sometimes does not coincide with the physiotherapist' opinion, who usually finds it too early. • The stigma associated with walking aids influences the patient's receptivity and the neurologist's decision to suggest its use. • Patients face the recommendation as a defeat and with frustration.

#### Advanced-Stage Group

None of the patients used walking aids regularly. They see it as potentially helpful, but they try to postpone its use as much as possible, through medication adjustments. Advanced-stage patients have doubts about their usefulness due to the presence of motor fluctuation (in the “ON” medication state, they do not think the need for this kind of help), postural instability, and upper limb problems (which in their perspective hampers its use). Patients who have already used walking aids did it on their initiative, without medical advice, training, or adaptation. The occurrence of falls, the feeling of insecurity, and the resistance to use again on medical recommendation after a bad experience were mentioned.

Due to the lack of experience with walking aids, patients did not feel being able to define their key characteristics.

#### Physiotherapists

To physiotherapists, a threat to patient's safety (e.g., increased postural instability or the occurrence of falls) determines the recommendation of walking aids. According to them, this type of help is not always well-received. Sometimes, it is perceived as something negative, as a sign of disease progression and of greater dependence. The fear of falling was mentioned as a factor that facilitates its use. It was also referred that some patients start using walking aids too early, without clinical recommendation. Physiotherapists stressed the need to adapt walking aids to patient characteristics and needs and the importance of a supervised period of training. General key characteristics were not mentioned.

#### Neurologists

In the neurologist's perspective, walking aids should be prescribed according to the patient's clinical characteristics. Neurologists referred to approach this topic during consultations but to leave the decision to the physiatrist or physiotherapist, since they are more prepared to make a formal recommendation. It was also mentioned that their opinion about the need for this type of aids does not always coincide with the physiotherapist's opinion.

In neurologist's perspective, the use of walking aids is often seen by patients as a loss of autonomy and never as a gain in FM, due to the stigma associated with its use. They referred the need to approach the topic carefully and that patient's reactions are usually defeat, frustration, or taking offense. Neurologists emphasize the importance of a training period. They also recognized that the recommendation of walking aids is sometimes hindered by their own prejudice in relation to this type of aids. This sometimes makes them postpone its recommendation, more than would be desirable.

Neurologists believe that the characteristics of a walking aid should be indicated by a physiatrist or physiotherapist.

## Discussion

Ten patients and 10 health professionals participated in the focus groups. All patients were assessed in “ON” state medication. Patients in the advanced-stage group were all recruited from the DBS surgery waiting list, whereby although younger and with a lower score in MDS-UPDRS part III (motor score), had a more disabled type of PD.

### The Concept of Functional Mobility

Although none of the groups has provided a definition that fits the proposed definition perfectly, the FM concept seems to be well-understood by patients and professionals and reflects patient's daily life difficulties and disease progression.

Early-stage patients and neurologists seem to be more focused in the component of mobility, whereas advanced-stage patients and physiotherapists highlight more the component of function. In reality, FM is a specific type of mobility that requires displacement and the engagement in tasks and activities at home, work, and in the community (Table 2).

In the neurologist's opinion, the FM concept should not be limited by the need for displacement but is defined as the ability to do what one proposes. This idea seems to be present in other groups since references to functional tasks like dressing, shaving, or drinking water were frequent. However, the existence of a displacement is a key component of the concept. FM is the ability of a person to move and is operationalized by the assessment of gait, balance, and transfers during the performance of a functional task (4, 5). This requires displacement and excludes all types of upper limb mobility. Also, this suggestion of a broader concept of FM falls into the definition of mobility [i.e., as “moving by changing body position or location or by transferring from one place to another, by carrying, moving or manipulating objects, by walking, running or climbing, and by using various forms of transportation.” (6)], whereby its adoption would be to give a new name to an existing and already established concept (Tables 2, 5).

**Table 5:** The definition of FM and related concepts (6–8).

Functional mobility	A person's physiological ability to move independently and safely in a variety of environments in order to accomplish functional activities or tasks and to participate in the activities of daily living, at home, work and in the community.
Mobility	The ability to move by changing body position or location or by transferring from one place to another, by carrying, moving or manipulating objects, by walking, running or climbing, and by using various forms of transportation.
Functioning	The individual's ability to execute a task or an action of daily life activities. Refers to all body functions, activities and participation.
Disability	A physical, mental, cognitive, or developmental condition that impairs, interferes with, or limits a person's ability to engage in certain tasks or actions or participate in typical daily activities and interactions.
Independence	The ability to carry out activities that support one's own lifestyle and to control the care given by others.
Autonomy	Self-rule that is free from both controlling interference by others and from limitations, such as inadequate understanding, that prevent meaningful choice.

The way the different groups described the concept seems to reflect their personal knowledge and experience on FM limitations. While early-stage patients and neurologists seem to see it as a minor or distant problem, advanced-stage patients and physiotherapists face it as a current and major problem.

Neurologists also suggest the use of FM as an outcome that better reflects the patient's perception and needs regarding their overall health status. This seems to go in line with the idea previously published that although the assessment of specific disease-related outcomes (e.g., tremor and rigidity) is important, evaluating functional limitations is crucial to get a better idea of a PD patient's disability profile (3). Future studies should explore if and how discrepancies about the concept between patients in different stages and health professionals affect the FM problem management.

### The Impact of FM Limitations in Patient's Lives

Once more, the perspective of early-stage patients seems closer to neurologists and that of advanced-stage patients to physiotherapists. For advanced-stage patients and physiotherapists, with a closer experience of FM limitations, it was easier to describe its interference in daily activities and its social impact and to mention strategies to overcome them (Table 6).

**Table 6:** The differences and similarities in the opinions of patients and health professionals.

**Shared perspectives**	
• FM is related to the ability to move and perform tasks in daily life	
• FM is impaired in PD	
• There are different degrees of limitation, associated with disease progression	
• Patients look for strategies to minimize FM limitations	
• Exercise, cognitive training are efficacious strategies to deal with FM limitations	
• There is a stigma associated with the use of walking aids	
**Different perspectives**	
**Patients**	**Health professionals**
**What is the impact of FM limitations on the patient's daily life?**	
• For early stage patients FM problems are mainly a problem of slower rhythm. • With the disease progression there is a clear perception of FM limitations, being the most limiting factor of activities of daily living • Advance stage patients feel ashamed for drawing others' attention	• First limitations: stand up from a chair, get out of the bed or from the car • Patients initially devalue the FM problems, then fell sadness and frustration. • Vary from patient to patient, according to lifestyle and tolerance with himself
**The use of walking aids**	
• From the perspective of early stage patients, walking aids are not necessary for PD. • For both early and advance patients FM problems can be solved with good management of the disease, prevention and education by a specialist • Patients try walking aids on their own initiative, without a previous training period. Falls occur. • For both early and advanced patients, the ability to complete a task successfully is more valuable than the time spent with it.	• The presence of imbalances and an increased risk of falling are the first warning signs for the need for walking aids. • Patients face the recommendation negatively. The fear of falling helps to accept the recommendation of a walking aid. • The choice of walking aid should be personalized, according to the patient's clinical characteristics. Physiotherapy sessions are important to a test and adapt to the walking aid that best suits the patient. • The time spent performing a task is also a concern.

The awareness of having a disease and the experience of limitations, even minor, in daily life lead patients to value more the ability to successfully complete a task, rather than the time needed to perform it (9, 10). This is noteworthy since one of the main reasons for being excluded from work and community environments is to be unable to move at an intensity and frequency that life requires (7). This goes in line with the idea of a previous paper on FM in PD, in which the author refers the superiority of perceived control above velocity (7, 11). As mentioned in the paper, the understanding of these determinants will help health professionals to have a more patient-centered intervention. In a time where personalized interventions are gaining relevancy, being aware of these aspects is crucial and may help to blur the differences between patients and neurologists and/or caregiver's perspectives.

It’s also relevant the social impact of the disease. Patients feel ashamed in public environments because of tremor and functional limitations, and little understood by friends because of the fluctuating aspect of the disease. Neurologist mentioned that the impact and degree of discomfort with FM limitations vary with the level of tolerance of patients. According to a 2017 cross-sectional study (12), the stigma of the disease and patient's emotional well-being affect not only the patients but also caregivers. In line with this, we hypothesize that a joint work from the psychology team with physiotherapy for teaching compensatory strategies may be useful to help patients dealing with FM limitations and to lessen the disease burden for patients and caregivers. It would be interesting to know in future studies the weight of the different motor and non-motor symptoms for PD patient's FM problems. This may help to optimize the management of these problems.

### The Use of Walking Aids

The stigma associated with the use of walking aids hinders its use by patients, in early and advances stage of the disease, and interferes with neurologist's recommendations. Although walking aids could allow for a more active lifestyle, the fact of being associated with disability prevent them from being considered as something that may enhance perceived control of their situation (7).

It is interesting to note the openness and acceptance of walking aids based on technological devices or in instruments that do not have the classic appearance of walking aids (e.g., Nordic sticks). It is also curious that even when patients suggest the development of technological walking aids, they do not seem to want to use them to be faster or to have a more active lifestyle, but to correct aspects that draws other's attention (posture, dyskinesias, and freezing).

Due to the size of our sample and the fact that all patients have the same nationality, we recognize that these results were influenced by cultural factors. We believe that the information generated here is important to highlight the relevance and usefulness of the concept of FM for PD management and research. However, we recommend conducting in the future a larger and multinational study.

## Conclusion

Functional mobility limitations were acknowledged by early-stage PD patients, representing an important limiting factor of daily activities and social participation for advanced-stage patients. The proposed concept of FM to be applied to PD seems to be well-understood by patients and health professionals and reflects the impact of disease progression in patient's lives. Although walking aids have the potential to increase patient's FM, they are seen as a sign of dependency; therefore, they are not well-accepted. Future bioengineering studies should focus on a technological solution and avoid the look of classical walking aids. We recommend the adoption of FM as an outcome, in clinical routine and research, as a strategy to get a better perception of patient's overall health status and to adopt a more patient-centered approach.

## Data Availability Statement

All datasets generated for this study are included in the article/Supplementary Material.

## Ethics Statement

The studies involving human participants were reviewed and approved by CNS Local Ethical Committee (Ref. 04-2018). The patients/participants provided their written informed consent to participate in this study.

## Author Contributions

RB-M conceptualized the study, executed the study and data analysis, and drafted and revised the manuscript. NG executed the study and data analysis and revised the manuscript. IL, MP, PC, RN, SD, AC, AV, PL, LG, MR, and MC executed the study and revised the manuscript. JF conceptualized the study and revised the manuscript. All authors contributed to the article and approved the submitted version.

### Conflict of Interest

LG is a consultant for Novartis and Sanofi-Genzyme, a board of adviser for GMP-Orphan, and is employed at the Department of Neuroscience and Mental Health, Neurology, Hospital de Santa Maria, CHULN, Lisbon, Portugal. JF is a consultant for Ipsen, GlaxoSmithKline, Novartis, Teva, Lundbeck, Solvay, Abbott, BIAL, Merck-Serono, and Merz; received grants from GlaxoSmithKline, Grunenthal, Teva, and Fundação MSD; and is employed at the Laboratory of Clinical Pharmacology and Therapeutics of Lisbon. The remaining authors declare that the research was conducted in the absence of any commercial or financial relationships that could be construed as a potential conflict of interest.
