# Supplementary material for: Patients and Health Professional's Perspective of Functional Mobility in Parkinson's Disease
Source: Front Neurol. 2020 Oct 27;11:575811. doi: 10.3389/fneur.2020.575811 (PMC7657224; doi:10.3389/fneur.2020.575811)
Supplement: Supplementary file 1 [file Data_Sheet_1.docx]

Appendix 1

**Focus groups script**

1. **Focus Groups with PD patients**

The researchers will meet the PD focus group subjects and pose the questions present on the first column of Table 1 while making sure they address the topics of column 2. This shall take up to 60 minutes (55 minutes to focus groups questions and 5 to close).

At the beginning of each interview, participants will be reminded of the purpose of the study and guaranteed confidentiality. Further, it will be told to show respect for others’ views and take turns in speaking. The participants will be encouraged to interact with each other, with the author intervening solely to keep the discussion on topic, and to encourage the more reserved members of the group to speak. In the beginning of the interview, after the opening question, the concept of functional mobility will be explained.

Functional mobility is the physiological ability of people to move independently and safely in a variety of environments in order to accomplish functional activities or tasks and to participate in activities of daily living (ADL), at home, work and in the community.

| Category | Questions | Make sure the participants address |
| --- | --- | --- |
| Opening questions | How long have you been diagnosed with Parkinson’s disease?  Do you maintain an active lifestyle?  What does the concept of functional mobility mean to you? | To introduce the topic of discussion and get people to start thinking and talking about their connection with the topic. |
| Impact of functional mobility limitations in patients’ life | Since disease onset did you feel more difficulties moving around to perform ADL, in home, work or during other social interactions? | - Onset of difficulties - Which ADLs |
|  | You think that this is a problem properly understood by health professionals, family and coworkers? | - Difficulties reporting these limitations and its impact - Impact in home, work or leisure activities - Social embarrassment or potential misunderstanding of difficulties |
|  | In your opinion which were the most efficacious strategies to help you copying with the functional mobility impairments? | The role of pharmacological and non-pharmacological interventions |
| The use of walking aids | When do you have an activity to perform, which aspect do you values most? (ability to perform correctly, the time needed, autonomy/perceived control, etc.) |  |
|  | Would you like to use walking aids to help you in your daily life tasks? | - Previous experiences with walking aids - Shortcomings of safety devices - Perceived control - Autonomy |
|  | In what situations would you consider using walking aids? | - Personal factors (e.g. to be more independent) - Activities that justify its use (e.g. possibility to work, to go for shopping, feeling of safety) |
|  | Which are the most important characteristics to adhere to walking aids? | Examples: dimensions, weight, adjustment, safety, durability, easy of use, comfort, effectiveness. |
| Ending questions | Thank you for your time today. Is there anything that you would like to say that I have not covered? | --- |

1. **Focus groups with health professionals**

The researchers will meet the health professionals group and pose the questions present on the first column of table while making sure they address the topics of column two. This shall take up to 60 minutes (55 minutes to focus groups questions and 5 to close).

At the beginning of each interview, participants will be reminded of the purpose of the study and guaranteed confidentiality. Further, it will be told to show respect for others’ views and take turns in speaking. The participants will be encouraged to interact with each other, with the author intervening solely to keep the discussion on topic, and to encourage the more reserved members of the group to speak. In the beginning of the interview, after the opening question, the concept of functional mobility will be explained.

Functional mobility is the physiological ability of people to move independently and safely in a variety of environments in order to accomplish functional activities or tasks and to participate in activities of daily living (ADL), at home, work and in the community.

| Category | Question | Make sure the participants address |
| --- | --- | --- |
| Opening questions | How long do you work with Parkinson disease patients?  What does the concept of functional mobility mean to you? | To introduce the topic of discussion and get people to start thinking and talking about their connection with the topic. |
| Impact of functional mobility limitations in patients’ life | When do you think that PD patients’ functional mobility limitations start? Which are the main problem related with functional mobility in daily life? | - Onset of difficulties - Which ADLs |
|  | How do you think that PD patients cope with functional mobility limitations?  Patients express easily this type of difficulties? | - Difficulties reporting these limitations and its impact - Impact in home, work or leisure activities - Social embarrassment or potential misunderstanding of difficulties |
|  | In your opinion which were the most efficacious strategies to help PD patients copying with the functional mobility impairments? | The role of pharmacological and non-pharmacological interventions |
| The use of walking aids | Which are the most important aspects when a PD patient has to perform an ADL? (ability to perform correctly, the time needed, autonomy/perceived control, safety, etc.) |  |
|  | When do you consider recommending the use of walking aids to a patient? | - Functional state of patients - Rehabilitation potential - Personal or professional demands |
|  | How do patients respond to the possibility of using a walking aid? | - Perceived control - Autonomy - Activities that justify its use (e.g. possibility to work, to go for shopping, feeling of safety) |
|  | Which are the most important characteristics to adhere to walking aids? | Examples: dimensions, weight, adjustment, safety, durability, easy of use, comfort, effectiveness. |
| Ending questions | Thank you for your time today. Is there anything that you would like to say that I have not covered? | --- |
