# Supplementary material for: Patients and Health Professional's Perspective of Functional Mobility in Parkinson's Disease
Source: Front Neurol. 2020 Oct 27;11:575811. doi: 10.3389/fneur.2020.575811 (PMC7657224; doi:10.3389/fneur.2020.575811)
Supplement: Supplementary file 2 [file Data_Sheet_2.docx]

Appendix 2

**Illustrative quotes of the topics approached during the focus groups**

| ***The concept of FM*** | |
| --- | --- |
| Early-stage patients | “Is what we do on a daily basis”  “Is whether or not we continue to have the same facility in carrying out activities we did before, at least we have the perception of having this type disease”  “Is the ability to move” |
| Advance-stage patients | “Autonomy for day-to-day”  “Don't need others”  "Is to be able to go out on the street without anyone noticing that I have Parkinson's"  “Is wanting to do things and it seems like I don't know how to do it”  “Is to get dressed and move in bed” |
| Physiotherapists | “It's a person's functionality… I can move my arm, but what is it for if I can't grab things, I can't eat”  "It is a movement that we have, which can be more or less limited, but which can be useful in our daily lives, and for the things that are important to us"  “Being able to move in a functional way”  "I move to a role"  “Work on mobility in order to guarantee some coordination afterwards for the function performed, because this is often what they are looking for” |
| Neurologist | “The ease of movement of patients”  "The ease, or the movement that is needed to perform a task or function"  “Transfers”  “In a broader sense, it is doing what they want regardless of how you do it”  "Functional mobility would be close to the WHO concept of disability as opposed to impairment, which is physical disability only" |
| ***The impact of FM limitations in patients’ life*** | |
| Early-stage patients | “Functional mobility is something that I never worried about until I had this thing (the disease)”  “My functional mobility is impaired”  “The rhythm in the bathroom (…) is slower”  “Our rhythm is different from what we had 10 years ago”  “Friends don't see the difficulty of buttoning; (…) My wife sees it.”  "The problem of the tremor, and not living together (...) people ask (...) I tell people that it is anxiety, and everything is under control, people remain in their ignorance."  “I think physical exercise is essential… don't stop!”  “If you do nothing with your brain you also lose it” |
| Advance-stage patients | “I have difficulty getting on and off a public transport… more to get off than to go up, if it is on a public transport standing up, everything is looking at me”  “When I call attention, I feel very ashamed (…) sometimes to prevent people from looking at me, I do something (…) I start running (…) my left toes start to close and I can't walk, so the only way to unlock it is to try to run or walk faster so your fingers don't close ”  “Some friends are aware of the disease, others are not… they start asking me questions about the disease that it is difficult for me to answer (…) it seems that they do not look very well at what I was and what I am."  “I removed the shower doors so that he had better mobility in and out of the bathtub” |
| Physiotherapists | “There is the phase of devaluing, in the beginning, and then the phase of frustration”  “They end up adjusting the situation… for example, I have a patient who, as he couldn’t turn around in bed, already lies in the position where he will stay all night. If he has several chairs at home, he sits on the tallest.” |
| Neurologist | “It depends on the patient, it depends on the patient's level of demand, if he is a designer or an architect right at the beginning of the illness, this interferes with his profession… if he is a person who is less demanding with himself, he tolerates much more the incapacity that goes by having"  "It depends on things as simple as whether he is right-handed or left-handed (...) for example the hand that is slow on the right and he is left-handed often comes to the doctor much later than vice versa"  “The patient has a tremor in his hand and does not say that it bothers him”  “The patient has many dyskinesias and that doesn't bother him”  “It is the patient himself who is managing to a point where he can no longer manage”  “Sometimes at the consultation we think he can't walk and he just wants to button his shirt, and for us it's a bit disconcerting, we will adjust the medication and get him going, this is an extreme, get him to do something else and he just wants to do this (…) you have to be methodical in the consultation to get this. ” |
| ***The use of walking aids*** | |
| Early-stage patients | “To perform a task”  “To complete successfully”  “Time is no longer a priority. When we realize that we have the disease, time is no longer a priority.”  “Having a sensor that would tell us “look, you're in the wrong position” or “straighten your back”, that is what my daughter says.”  "Parkinson's does not ask for these solutions"  “I don't need it for now”  "It depends a lot on the degree and the needs of each one (...) all this must be faced in a progressive way and according to the need of each one ... but at this moment I say no, give me idea that I don't need anything" |
| Advance-stage patients | “I was also told to walk with a cane, I don't use it because I'm ashamed.”  “I consider using it, but I don't know, so far I haven't decided yet”  "When I'm OFF (...) I think it will help not to fall, not to hit the walls of buildings"  "There were times when I staggered a lot and when I got up from a chair, sofa or something, I had to lean against the wardrobe, touch the furniture, and so it went right to the bathroom, for my initiative I took a crutch to see if it worked."  “We have to learn to walk with a cane, to know if the feet go first, if you go right, left ...”  “There are times when I need it but there are other times when I don't”  “I don't think this will help me much in my balance, because my lack of balance is such that there is a cane that works (…) I also have problems with my hand and arms”  “Walking aids don't give me the safety I need” |
| Physiotherapists | “Imbalances and the risks of falling are the first warning signs to think of a walking aid.”  “Only those who are afraid of falling will accept it well”  "There are people who use the walking aid early!" |
| Neurologist | "I think that physiotherapists are more competent than me to say if that patient benefits from having a walker or a wheelchair"  “Many times, the patients face the use of a walker or a wheelchair not as a gain in functional mobility, that is, but as a loss of autonomy associated with the stigma that a wheelchair has.”  "We are arriving a little late (…) Leads that many patients to have very serious complications. The risk for an 80-year-old patient of having a fractured femoral neck is never to be able to walk or sit again. It is going from being able to walk to being bedridden.”  “Patients are offended”  “It's a stigma, they think whoever gets to the chair doesn't get out of the chair”  “It is necessary to convince that the chair is a help and not a definitive thing.”  “I try to convince, (...) it takes a lot time of the consultation.”  “We have to assume that it is a stigma between us and people, so that we can change our attitude and build change” |
